# EXPLORING THE POTENTIAL OF VOICE INTERFACES FOR EMERGING TECHNOLOGIES: OPPORTUNITIES AND CHALLENGES

**DOI:** 10.1093/geroni/igac059.861

**Published:** 2022-12-20

**Authors:** Wendy Rogers, George Mois, Kenneth Blocker, Raksha Mudar

**Affiliations:** University of Illinois Urbana Champaign, Champaign, Illinois, United States; University of Illinois Urbana-Champaign, Urbana Champaign, Illinois, United States; University of Illinois Urbana-Champaign, Champaign, Illinois, United States; University of Illinois Urbana Champaign, Champaign, Illinois, United States

## Abstract

Successful human-technology interactions require that people be able to communicate their intentions to the technology. Emergent technologies such as virtual assistants, digital home assistant, smart phones, robots, and smart appliances are enabling voice activation and communication. This is potentially a positive trend as people often express a preference for using familiar methods of communicating with other people in their interactions with technologies. Presumably they expect these methods to be easier to learn and more effective. However, current voice technology interfaces are often dependent on specific language, cadence, and terminology that pose challenges for older adults to master. Moreover, age-related changes in voice characteristics, speech patterns, and the variability between individuals may lead to comprehension failures on the part of the technology. We have utilized research findings to develop implementation guidelines for use of voice interfaces with older adults. We also identify research gaps to provide a roadmap for future efforts.

